# Annotations

**Published:** 1909-12-11

**Authors:** 


### Annotations.

### Personal Cleanliness.

There is such a thing as a Cleaning of Persons Act, and it is a pity that it is not more generally known, or its clauses more generally enforced. Several inquests recently held have shown that among the poorer classes, especially of the type that finds night lodgings in the casualty wards, personal uncleanliness is the rule. In one instance, when the deceased was especially verminous and foul, the Coroner expressed the opinion that the above-mentioned Act should be made compulsory, and that the police should be given powers to take unclean loafers to local cleansing stations. This appears to us to be an excellent suggestion. At any rate, it is highly desirable that local sanitary authorities should be given power to deal with persons whose filthy condition makes them a danger to the community. Such individuals should be systematically disinfected and purified, and for that reason it is necessary that special sanitary stations should be established to deal with such cases. There is unlimited scope for the popularisation of personal hygiene, and the application of its principles in this country.

### Bread and the Baker.

Dr. Meredith Young, Medical Officer of Health for the Marylebone Division, draws attention in his latest annual report to the sanitary carelessness with which the average baker treats his bread. "It is a matter which may be verified in the streets of any town on any occasion," he remarks, "that bread by being carried about in baskets in which it is openly exposed to all the dust which is blowing about the roads, by being not infrequently dropped in the roads, and hastily wiped on the hand or clothes of the carrier, and so forth, is as regards the cleanliness of the crust bound to be a matter of public concern. I have communicated with the largest bread companies in London, and those who have taken the trouble to reply have admitted the importance of delivering bread to the public in a cleanly state; but beyond seeing that their vans and baskets are kept clean, they take no precaution to ensure its arrival at the table in a clean condition. It is stated that bread cannot be put into a wrapper while warm; again, in large businesses, as soon as it is cool, it must be loaded into vans for quick delivery. It is further stated that it would require a large staff and entail very great expense to wrap each loaf individually in paper, and, finally, that the public would probably not be prepared to pay any additional cost for bread so protected. Of the truth of some of these assertions I have very serious doubts." With this expression of opinion we must cordially agree. A little cleanliness apparently goes a long way with the average baker's boy, whose dirty glove ought to be an interesting study to the seriously-minded bacteriologist; but the fault is really the customer's. If the public demanded clean bread, cleanly handled, it would put a stop to the habits described by Dr. Young, and guarantee, at least, a weekly wash for the boy's glove.

### Medical Students and "Dr." Bodie.

On November 16 last some dozen medical students were fined at London police courts for small offences arising out of a sort of demonstration directed against the now notorious "Dr." Bodie. These escapades are not to be defended; and, in pretty recent times, at any rate, their perpetrators were not drawn from the best elements among medical scholars. The comment of a Magistrate, too, "as bad as the Suffragettes," is fair enough. But, while saying just as much as befits a professional journal on the subject of the ethics of breaches of the peace as a means of propaganda, we might point out that the remark can be interpreted not altogether unfavourably. Of late years little *émeutes* like the present one have nearly always been provoked. The false and canting inscription under a certain statue at Battersea is an instance in point. At those wells of pure learning, the ancient Universities, on the contrary, it is fairly common for private property to be wantonly destroyed, and distinguished persons mobbed, under no provocation whatever. The London medical student, however, resembles rather the bear in the well-known story, whose ferocity was shown in defending itself from attack. And if the exuberantly developed sense of solidarity common in youth finds indiscreet manifestations there is at any rate some excuse to be advanced.

### The Late Charles R. B. Keetley, F.R.C.S.

It was with feelings of profound sorrow that we heard of the death of Charles Robert Bell Keetley, F.R.C.S., at Brighton, on Saturday, December 4. A man of many parts, with few, if any, enemies, he held with hoops of steel life-long hosts of friends. Not only eminent as a surgeon, his chief charm to his friends was his wonderful personality and character. Original in everything, whether it was a question in surgery, politics, art, or the common affairs of life, his talk had a force and charm which many of his friends and colleagues will miss sadly. Staunch in his friendships, were they high or humble, he was always the same. Forgetful of many things likely to advance his worldly prospects, he never, in the recollection of the writer, forgot little and great acts of kindness. Hasty at times, all soon blew over, and he was never known to bear malice against any human being. He was in the best sense of the term "the strong man armed." Never at a loss what to do in the most difficult operations, his great knowledge and judgment inspired in all who knew him a deep and lasting admiration. His delightful humour, never failing at the right moment, was of the purest, with no tinge of coarseness, untainted with sarcasm or spite. A strong character, remarkable for the keen love and affection he had the wonderful gift of inspiring in all his many friends.

